# Cholesterol-Lowering Action of a Novel Nutraceutical Combination in Uremic Rats: Insights into the Molecular Mechanism in a Hepatoma Cell Line

**DOI:** 10.3390/nu12020436

**Published:** 2020-02-09

**Authors:** Maria Giovanna Lupo, Noemi Biancorosso, Elisa Brilli, Germano Tarantino, Maria Pia Adorni, Greta Vivian, Marika Salvalaio, Stefano Dall’Acqua, Stefania Sut, Cédric Neutel, Haixia Chen, Alessandro Bressan, Elisabetta Faggin, Marcello Rattazzi, Nicola Ferri

**Affiliations:** 1 Dipartimento di Scienze del Farmaco, Università degli Studi di Padova, 35131 Padova, Italy; mariagiovanna.lupo@phd.unipd.it (M.G.L.); noemi.biancorosso@unipd.it (N.B.); greta.vivian@gmail.com (G.V.); marika.salvalaio@unipd.it (M.S.); stefano.dallacqua@unipd.it (S.D.); stefania_sut@hotmail.it (S.S.); 2 PharmaNutra S.p.A., 56122 Pisa, Italy; e.brilli@pharmanutra.it (E.B.); g.tarantino@pharmanutra.it (G.T.); 3 Dipartimento di Scienze degli Alimenti e del Farmaco, Università di Parma, 43100 Parma, Italy; mariapia.adorni@unipr.it; 4 Laboratory of Physiopharmacology, Research Unit GENCOR, University of Antwerp, 2610 Antwerp, Belgium; cedric.neutel@uantwerpen.be; 5 Tianjin Key Laboratory for Modern Drug Delivery & High-Efficiency, School of Pharmaceutical Science and Technology, Tianjin University, Tianjin 300072, China; chenhx@tju.edu.cn; 6 Dipartimento di Medicina, Università degli Studi di Padova, 35121 Padova, Italy; alessandro.br94@gmail.com (A.B.); elisabetta.faggin@unipd.it (E.F.); marcello.rattazzi@unipd.it (M.R.); 7 Medicina Generale I^-Cà Foncello Hospital, 31100 Treviso, Italy

**Keywords:** MK-7, cholesterol, PCSK9, uremic, mevalonate pathway

## Abstract

Appropriate nutraceutical combinations may represent a valid approach to prevent vascular calcification associated with chronic kidney disease (CKD). In the present study, we tested the effect of a new nutraceutical combination named RenaTris^®^, containing MK-7, magnesium carbonate, and Sucrosomial^®^ Iron, on vascular calcification in uremic rats. Rats were randomly divided into three groups, i.e., control (high-phosphate diet), uremic (high-phosphate diet containing 0.5% adenine), and supplemented uremic diet (0.5% adenine, MK-7, magnesium carbonate, and Sucrosomial^®^ Iron). After six weeks, sera and vascular calcification were examined. The uremic diet increased creatinine and phosphate levels and induced extensive vascular calcification. The uremic condition also induced a mild hypercholesterolemic condition (+52% of total cholesterol; *p* < 0.05). The supplemented uremic diet did not reduce creatinine, phosphate levels, or vascular calcification, however, we observed a significant hypocholesterolemic effect (−18.9% in supplemental uremic vs. uremic diet; *p* < 0.05). Similar to simvastatin, incubation of cultured human hepatoma cells (Huh7) with MK-7 significantly reduced cholesterol biosynthesis (−38%) and induced 3-hydroxy-3-methyl-glutaryl-CoA (HMG-CoA) reductase and low-density lipoprotein receptor (LDLR) at both mRNA and protein levels. The effect of MK-7 on LDLR was counteracted by the co-incubation with squalene. Unlike simvastatin, MK-7 reduced PCSK9 in Huh7. These results indicated that the new nutraceutical combination significantly impacts cholesterol metabolism and its supplementation may help to control mild hypercholesterolemic conditions in CKD patients.

## 1. Introduction

Chronic kidney disease (CKD) represents one of the most important and independent risk factors of a variety of cardiovascular diseases, including heart failure [1], stroke [2], peripheral artery disease [3], coronary heart disease [4], and atrial fibrillation [5]. CKD is frequently associated with dyslipidemia, low-grade inflammation, and vascular calcification [6,7]. The entire plasma lipidome changes with the severity of CKD [8]. Patients with CKD exhibit proatherogenic dyslipidemia similar to that of patients with type 2 diabetes, with low high-density lipoprotein (HDL) levels and high lipoprotein (a) (Lp(a)) levels [9,10,11]. The influence of altered lipid metabolism in atherosclerotic artery disease observed in patients with kidney failure and CKD is not clear [12]. However, additional key players in the disease include calcification inhibitors (e.g., fetuin-A and matrix Gla protein), promoters (e.g., hyperphosphatemia), calcium phosphate product, parathyroid hormone, and leptin [12,13].

Matrix Gla protein (MGP) is a powerful inhibitor of tissue calcification. Active MGP is a strong endogenous inhibitor of soft tissue calcification [14]. Vitamin K is an essential co-factor for the carboxylation of MGP, turning inactive uncarboxylated MGP into active MGP [15].

Vitamin K occurs in two dietary forms, i.e., vitamin K1 (phylloquinone) and vitamin K2 (menaquinones). Vitamin K1 is mainly found in green leafy vegetables and vitamin K2 is mainly found in fermented foods such as cheese and “natto”, a Japanese soybean product [16]. Liver is also a rich source of menaquinones [17]. More than 12 different types of MK-n have been identified, from MK-4 to MK-15, where “n” indicates the number of unsaturated isoprenoid residues linked to the menaquinone. In particular, MK-7 is produced by bacteria and shows a favorable pharmacokinetic profile compared to MK-4, including a longer half-life time and higher bioavailability. The most common MK in humans is the short-chain MK-4, the only MK produced by systemic conversion of phylloquinone to menaquinones [18]. Vitamin K is a non-polar molecule; after its intestinal absorption, vitamin K is solubilized by bile salts and pancreatic juice and packaged into chylomicrons, which are secreted into the lymphatic system [19].

CKD patients frequently suffer from subclinical vitamin K deficiency [20], resulting in increased plasma levels of inactive uncarboxylated MGP protein, a condition that may be associated with an increased risk of cardiovascular disease (CVD) [21] and mortality in patients with CKD [22,23]. This deficiency might be caused by exhaustion of vitamin K due to its high requirement by vitamin K-dependent proteins to inhibit calcification or due to dietary recommendations for CKD patients, such as a diet low in potassium (fewer leafy green vegetables rich in K1) and phosphate (fewer dairy products rich in K2) [24].

From this evidence, a nutritional intervention aimed at restoring vitamin K deficiency in CKD patients may represent a valid approach to slow the vascular calcification process and CVD risk. Recently, Scheiber et al. demonstrated a protective role of high-dose of vitamin K7 (100 µg/g diet) supplementation in a rat model of vascular calcification [25]. In the present study, we investigated the effect of diet supplementation with the components of RenaTris^®^ on vascular calcification in a uremic rat model. The supplemented diet did not reduce calcium deposition in the medial arterial wall, but a significant reduction in cholesterol plasma levels was observed. MK-7 demonstrated inhibition of cholesterol biosynthesis and modulated cholesterol metabolism-related genes in human hepatocarcinoma cell lines. Taken together, the present study identified a novel nutraceutical formulation, RenaTris^®^, with potential hypocholesterolemic actions.

## 2. Materials and Methods

### 2.1. Animals and In Vivo Experimental Protocol

This investigation conformed to the European Commission Directive 2010/63/EU and was granted approval by the “Direzione Generale della Sanità Animale e dei Farmaci Veterinari” of the Italian Ministry of Health (278/2017-PR). Male Sprague–Dawley rats (*n* = 33; Charles River laboratories) were fed a standard diet for 7 days. On day 1, they were randomly subdivided into three groups of 11 rats each, with each group fed one of the specific diets for 6 weeks (until day 42). The control group was fed the high phosphate diet (1.2% phosphate, 19% protein), the uremic group was fed a diet containing 0.5% adenine, high phosphate (1.2%) and low protein (4.5% protein), and the treated group was fed the same diet as the uremic group plus supplementation with MK-7 (3.5 µg/g of diet), MgCO_3_ (3.7 µg/g of diet), and Sucrosomial^®^ Iron (1 mg/g of diet), which was termed a supplemented uremic diet.

At death, the blood and aorta were collected. Sera were separated by centrifugation at 5000 g and stored at −80 °C until analysis. The aortas were dissected into two samples; the first were immersed in formalin for histological analysis, and the second were stored at −80 °C until required for later analysis. Serum concentrations levels of phosphate, creatinine, and iron were measured using an automated analyzer (Azienda Ospedaliera di Padova, Padova, Italy). The serum total cholesterol was determined by colorimetric assay (ABX Penta, cholesterol assay).

The examination of arterial medial calcification was performed on paraffin-embedded aortas that were deparaffinized and processed for von Kossa staining using the standard method. To quantitatively evaluate the degree of aortic medial calcification, frozen aortic tissues were weighed and hydrolyzed in 1 mL of 0.6 N hydrochloride acid for 24 h. The Ca^2+^ content of the supernatant was determined using commercially available calcium kits (Chema Diagnostica, Italy) and normalized to wet tissue weight (µg/mg wet weight).

### 2.2. Reagents

Eagle’s minimum essential medium (MEM), trypsin-EDTA, penicillin, streptomycin, sodium pyruvate, L-glutamine, nonessential amino acid solution, fetal calf serum (FCS), plates, and Petri dishes were purchased from EuroClone. Squalene was purchased from Sigma-Aldrich (St Louis, MO, USA). RenaTris^®^ capsules (500 mg) and MK-7 powder was supplied by PharmaNutra (Pisa, Italy), the contents of which were dissolved in 1.5 mL ethanol; the insoluble portion was separated by centrifugation. The supernatant was then evaporated under nitrogen flux and redissolved in 36 µL of ethanol in order to obtain a final concentration of 1.5 × 10^−3^M of MK-7. The final concentration of MK-7 was added to the cultured media was 1.8 µM. Simvastatin was dissolved in a physiological solution at 50 mM. Inorganic phosphate (Pi) solution was prepared by titrating 100 mM Na_2_HPO_4_ solution with 100 mM NaH_2_PO_4_ solution up to a pH of 7.4. All of the stock solutions were filtered through a 0.22 µM filter and stored at −20 °C. The Pi solution was stored at 4 °C.

### 2.3. Quantification of MK-7 Plasma Levels by LC-DAD-ESI-MS

For the analysis of the MK-7 plasma levels, 300 µL of plasma was diluted with the physiological solution and 300 µL of ethyl acetate was added and vortexed. The samples were centrifuged at 13,000 rpm for 15 min. The upper layer was collected and a second extraction was performed on the aqueous layer with another 300 µL of ethyl acetate. The organic layer was concentrated to dryness under nitrogen flow. The residue was dissolved in methanol (200 µL) and injected in the LC-MS/MS system. A Varian Triple Quadrupole model 320 with APCI source was used for analysis. For the chromatographic separation, an Agilent 1260 series liquid chromatography (LC) was employed with a binary pump using isopropanol and a mixture of methanol and water with 2% formic acid (19:1). The elution was performed in isocratic conditions using 50% of each eluent and a flow rate of 400 µL/min. A spectrometer was used in positive ion mode for the MK-7 transitions 649.6 > 227.2. A standard solution of MK-7 was used to construct a calibration curve in the range of 100–0.005 µg/L. Limit of detection (LOD) was assessed at 0.5 ng/mL.

### 2.4. Cell Cultures

Human hepatic cancer cells (Huh7) and human aortic smooth muscle cells were cultured in MEM supplemented with 10% FCS, L-glutamine, sodium pyruvate, nonessential amino acids, and penicillin/streptomycin at 37 °C in a humidified atmosphere of 5% CO_2_ and 95% air. For the experiments, the cells were seeded in MEM/10% FCS, and then the treatments were continued for an additional 24 h and 72 h for RNA and protein expression analysis, respectively. The final concentration of ethanol did not exceed 1% v/v and the same amount of solvent was added to all of the experimental points.

### 2.5. In Vitro Calcification Assay

The human aortic smooth muscle cells were treated for 7 days with inorganic phosphate (Pi) (2 mM) to induce calcification in the presence or absence of 0.03% or 0.06% of the supplement combination. At the end of the experimental period, the calcium levels were measured via alizarin red S (ARS) staining. Briefly, mineralization was assessed by extracting the calcified mineral at a low pH, neutralizing with ammonium hydroxide, and via colorimetric detection at 405 nm.

### 2.6. Cholesterol Biosynthesis Assay

The cells were cultured in 12-well plates. After 24 h, the cells were treated with increasing concentrations of MK-7 (1.9, 3.8, 7.5, and 15 µM) in 0.2% Bovine Serum Albumin (BSA)-containing medium for 24 h. The synthesis of cholesterol was then determined by measuring the incorporation of radioactive acetate into total cellular sterols [26]. After incubation with [2-^14^C] acetate (2 μCi/mL) in the presence or absence of MK-7 for 24 h, the cell monolayers were washed with phosphate-buffered saline and digested with 0.1 M NaOH overnight. Aliquots were saponified at 60 °C for 1 h in alcoholic KOH after the addition of 1,2-^3^H-cholesterol as an internal standard (10^5^ cpm/sample). The unsaponifiable material was extracted with low-boiling point petrol ether and counted for radioactivity. To evaluate the incorporation of labeled acetate into cellular sterols, these were separated from the unsaponifiable fraction via thin-layer chromatography using petroleum ether (boiling point: 40–60 °C)/diethyl ether/acetic acid (70:30:1). Radioactivity was measured by liquid scintillation counting. The data were expressed as counts per minute of [2-^14^C] acetate incorporation into total cellular sterols per milligram of protein.

### 2.7. Reversetranscription and Quantitative PCR (RT-qPCR)

Total RNA was extracted from cultured cells using the iScript^TM^ RT-qPCR Sample Preparation Buffer (Bio-Rad, Hercules, CA, USA) cDNA synthesis preparation reagents (Bio-Rad), according to the manufacturer’s instructions [27]. Reverse transcription–polymerase first-strand cDNA synthesis was performed using the Maxima First Strand cDNA Synthesis Kit (Thermo Scientific, Rockford, IL, USA). qPCR was then performed using the PowerUp^TM^ SYBR^TM^ Green Master Mix (Thermo Scientific) and specific primers for the selected genes. The primer sequences used for qPCR analysis are shown in Table 1. The analyses were performed with the Mx3000P qPCR System (Agilent), with under cycling conditions of 95 °C for 2 min, 95 °C for 15 sec, and 60 °C for 1 min for 40 cycles.

Total RNA was isolated from rat abdominal aortas using the TRI reagent^®^ protocol (Sigma-Aldrich) according to the manufacturer’ instructions. RNA purity and concentration were evaluated by spectrophotometry using a NanoDrop ND-2000 (Thermo Fisher Scientific).The levels of MGP transcripts were quantified by real-time PCR. Total RNA (1 μg) was retrotranscribed in cDNA using an iScript Reverse Transcription Supermix kit (Bio-Rad). qPCR was performed on 25 ng of each cDNA sample using SsoFast Eva Green Supermix (Bio-Rad) with a CFX96 thermocycler (Bio-Rad). The sequences of primers used in real-time PCR are shown in Table 1.

The data were expressed as Ct values and used for relative quantification of targets with ^∆∆^Ct calculations. The ^∆∆^Ct values were determined by multiplying the ratio value between the efficiency of specific primers and housekeeping 18S. The efficiency was calculated as ((10^(-1/slope))-1)*100.

### 2.8. Western Blot Analysis

Cells were washed twice with Phosphate Buffer Saline (PBS) and lysed with a solution of 50 mM Tris pH 7.5, 150 mM NaCl, and 0.5% Nonidet-P40, containing protease and phosphatase inhibitor cocktails (Sigma-Aldrich) for 30 min on ice. Protein samples (20 µg) and a molecular mass marker (Thermo Scientific) were separated using 4%–12% SDS-PAGE (Bio-Rad) under denaturation and reduction conditions. The protein samples were then transferred to a nitrocellulose membrane using the Trans-Blot^®^ Turbo™ Transfer System (Bio-Rad). The membranes were washed with Tris-buffered saline-Tween 20 (TBS-T) and nonspecific binding sites were blocked in TBS-T containing 5% nonfat dried milk for 60 min at room temperature. The blots were incubated overnight at 4 °C with a diluted solution (5% nonfat dried milk) of the following human primary antibodies: anti-PCSK9 (mouse monoclonal antibody, abcam ab84041; dilution 1:1000), anti-LDLR (mouse monoclonal antibody, Millipore clone 2H7.1; dilution 1:1000), anti-HMG-CoA reductase (rabbit polyclonal antibody, GeneTex; dilution 1:1000), and anti-α-tubulin (mouse monoclonal antibody, Sigma clone DM1A; dilution 1:2000). The membranes were washed with TBS-T and exposed for 90 min at room temperature to a diluted solution (5% nonfat dried milk) of the secondary antibodies (peroxidase-conjugate goat anti-rabbit and anti-mouse, Jackson ImmunoResearch). Immunoreactive bands were detected by exposing the membranes to Clarity^TM^ Western Enhanced ChemiLuminescence (ECL) chemiluminescent substrates (Bio-Rad) for 5 min, and images were acquired with a Azure c400 Imaging System (Aurogene) [28]. The densitometric readings were evaluated using ImageLab^TM^ software (Bio-Rad), as previously described.

### 2.9. ELISA

Conditioned media were cleared by centrifugation (15,000 rpm for 10 min at 4 °C) and stored at −20 °C. The amount of PCSK9 was then quantified using ELISA (R&D System) according to the manufacturer’s instructions and as previously described [10].

### 2.10. LDL-Isolation and Labeling

Total LDL (d > 1.019 < 1.063 g/mL) was isolated by ultracentrifugation at 4 °C from human plasma. To remove EDTA excess, the LDL samples were transferred to dialyzing tubes and dialyzed in physiological solution (0.9% NaCl in deionized water) at 4 °C, with the solution being changed three times (at 4 h, 48 h, and 48 h). The purified LDL was sterilized using a 0.22-µm filter and stored at 4 °C. The protein content was evaluated using a Bicinchoninic Acid Assay (BCA) assay [29], using BSA as the standard. For the labeling, LDL was incubated with fluorescent dye, DiO (250 µg DiO/mg LDL protein) for 18 h at 4 °C. The LDL–DiO sampled were passed through a Sephadex G25 column (PD10) with 0.01% PBS–EDTA (pH 7.4) to remove any unbound DiO [30,31].

### 2.11. Fluorescent LDL Uptake Cell-Based Assay

Huh7 cells were seeded in 6-well tray (3 × 10^5^ cells/well in a complete medium). After 24 h of treatment in 0.4% FCS media, cells were incubated with 10 µg/mL of LDL-DiO (3,3′- dioctadecyloxacarbocyanine). After 3 h of incubation at 37 °C, the cells were resuspended in PBS and analyzed by flowcytometry analysis (FC-500, Beckman Dickinson).

### 2.12. Statistical Analysis

Statistical analysis was performed using Prism statistical analysis package Version 5.01 (GraphPad Software, San Diego, CA, USA). When possible, *p* values were determined by Student’s t test. Otherwise, the differences between the treatment groups were evaluated by one-way ANOVA followed by Bonferroni’s multiple comparison post-hoc test. A probability value of *p* < 0.05 was considered to be statistically significant.

## 3. Results

### 3.1. Effects of MK-7, MgCO_3_, and Sucrosomial^®^ Iron on Vascular Calcification

**Figure 1:**
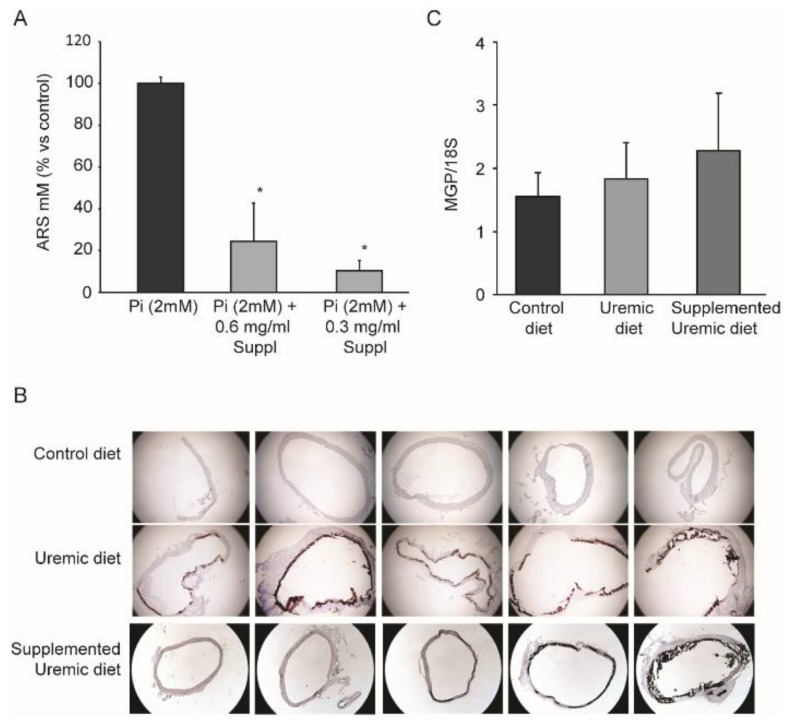
Effect of MK-7 in combination with magnesium carbonate and Sucrosomial^®^ Iron on vascular calcification. (**A**) In vitro calcification of vascular smooth muscle cells was induced by 2 mM of inorganic phosphate (Pi). The effect of the supplement combination (Suppl.) was tested at 0.3 and 0.6 mg/mL. Extracellular calcium was quantified by alizarin red S (ARS) staining. (**B**) Representative photomicrographs of abdominal aortas stained using the von Kossa method in control (upper panels), uremic (middle panels), and supplemented uremic diets (lower panel). The images are from 5 of 11 rats examined for each group. Although there was some variability between the rats, the induction of vascular calcification was observed by the brown/black staining in the tunica media of the uremic rats compared to the controls. The supplemental diet did not significantly affect the deposition of calcium. (**C**) Matrix Gla protein (MGP) mRNA expression from rat aortas determined by qRT-PCR. Student’s *t*-test: * *p* < 0.05 vs. Pi (2.0 mM).

To test the possible effects of a nutraceutical combination containing MK-7, magnesium carbonate, and Sucrosomial^®^ Iron on vascular calcification, we performed a series of experiments with vascular smooth muscle cells exposed to high concentrations of phosphate (2 mM) for seven days. As shown in Figure 1A, the phosphate induced a massive accumulation of inorganic calcium in the cultured media, as determined by alizarin red S (ARS) staining. The addition of the supplement combination (0.3 mg/mL and 0.6 mg/mL) significantly reduced smooth muscle cell calcification. These results prompted us to test the effects in an in vivo model of vascular calcification. Thus, to recapitulate the clinical condition of vascular calcification and altered lipid metabolism associated with CKD, we adopted an established in vivo model based on a diet supplementation of 0.5% adenine, which induced 70 ÷ 80% damage to kidney tissue, including fibrosis, increased blood urea nitrogen and plasma creatinine, azotemia, and abnormal electrolyte metabolism [32,33].

As expected, six weeks of a uremic diet significantly increased creatinine and phosphate levels, which was also associated with a minor reduction in iron plasma levels (Table 2). Interestingly, the uremic condition also induced mild hypercholesterolemia (+52% of total cholesterol; *p* < 0.05). The group of rats fed the uremic diet supplemented with MK-7, MgCO_3_, and Sucrosomial^®^ Iron showed similar altered levels of creatinine and phosphate with a nonsignificant increase in plasma iron levels (Table 2). Surprisingly, the supplementation significantly reduced total cholesterol plasma levels compared to the rats fed the uremic diet (−18.9%; Table 2).

The hyperphosphatemia status determined extensive vascular calcification in the tunica media of the aorta, as visualized by von Kossa staining (Figure 1B). Biochemical determination of the Ca^2+^ content in the aorta clearly demonstrated that the uremic condition strongly induced vascular calcification, and that the addition of MK-7, MgCO_3_, and Sucrosomial^®^ Iron did not alter this pathological condition (Table 2). In addition, we did not observe any significant variation in MGP mRNA levels from the total aortic RNA (Figure 1C).

Therefore, although the nutraceutical approach in our experimental model did not affect the vascular calcification, the reduction in cholesterol plasma levels captured our attention. Among the supplemental components added to the diet, MK-7 may have impacted the cholesterol homeostasis. Importantly, LC-MS/MS determination of MK-7 plasma levels demonstrated that significantly higher levels were observed in the rats fed the supplemental uremic diet than those fed the uremic diet (4.2 ± 3.5 ng/mL vs. 11.9 ± 7.9 ng/mL, respectively; *p* < 0.05).

### 3.2. Inhibition of Cholesterol Biosynthesis in the Hepatic Cell Line

**Figure 2:**
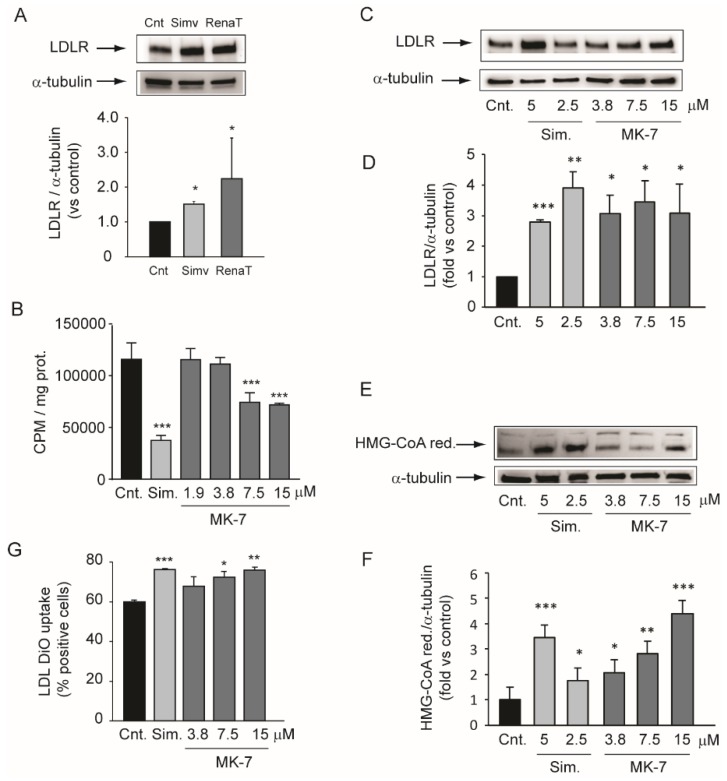
Effect of MK-7 on cholesterol synthesis and low-density lipoprotein (LDL) uptake. (**A**) Human hepatoma cells were incubated with simvastatin (5 µM) and ethanol extract of RenaTris^®^ (RenaT), as indicated in the Materials and Methods section. After 24 h, total protein extracts were prepared and low-density lipoprotein receptor (LDLR) expression was determined by Western blot analysis. α-tubulin was used as the loading control. (**B**) Human hepatoma cells were cultured in minimum essential medium (MEM) containing [2-^14^C]-acetate in the presence or absence of indicated concentrations of MK-7 or simvastatin (5 µM) as the positive control. [2-^14^C]-acetate incorporation was used to assay cholesterol biosynthesis 24 h after addition to cells. Each point represents the mean ± SD of triplicate dishes. (**C**,**E**) Human hepatoma cells were incubated for 24 h with indicated concentrations of simvastatin and MK-7. LDLR and HMG-CoA reductase protein expression were evaluated by Western blot analysis. α-tubulin was used as the loading control. (**D**,**F**) Densitometric readings were evaluated using the ImageLab^TM^ software. (**G**) Cells were seeded in MEM/10% fetal calf serum (FCS) and incubated the next day with MEM/0.4% FCS in the presence or absence of MK-7 and simvastatin (5 µM). After 24 h, 10 µg/mL of LDL-DiO was added to the cultured media and the fluorescence intensity was determined by flowcytometry analysis after 3 h of incubation. Data are given as the mean ± SD of triplicate dishes. Differences between treatments were assessed by Student’s *t* test. * *p* < 0.05 vs. control; ** *p* < 0.01 vs. control; *** *p* < 0.001 vs. control. Sim.: simvastatin.

To further explore the hypocholesterolemic effect of the supplemental diet, we set up a series of experiments using the human hepatic cell line. We first determined the effect of the ethanol extract from the nutraceutical combination. As shown in Figure 2A, 24 h of exposure to the nutraceutical extract significantly induced LDLR (+2.2 ± 1.1 fold) in Huh7 cells. Simvastatin was utilized as a positive control. We then extended this evidence by using MK-7 as a single active agent.

Incubation of Huh7 with 5 µM simvastatin for 24 h reduced the ^14^C-acetate incorporation into cholesterol by about 70% (Figure 2B). Under the same experimental conditions, MK-7 inhibited the cholesterol biosynthesis by 36% and 38% at 7.5 and 15 µM, respectively (Figure 2B). Next, we evaluated the effect of MK-7 on LDLR and HMG-CoA reductase levels. Similar to simvastatin, MK-7 increased the protein expression of both LDLR and HMG-CoA reductase in the Huh7 cell line (Figure 2CE). These effects were observed at the same concentration range as those that reduced cholesterol biosynthesis, with a maximal effect between the concentrations of 7.5 and 15 µM (Figure 2C–F). More importantly, MK-7 improved the capability of Huh7 cells to capture fluorescent-labeled LDL (LDL-DiO) (Figure 2G).

**Figure 3:**
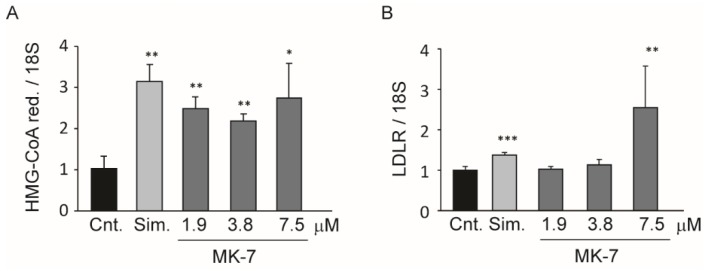
Effect of MK-7 on HMG-CoA reductase and LDLR mRNA expression. Cells were seeded in MEM/10% FCS and incubated the day after with MEM/10% FCS in the presence or absence of simvastatin (40 µM) or indicated concentrations of MK-7. After 24 h, total RNA was prepared and mRNA levels of HMG-CoA reductase (**A**) and LDLR (**B**) were determined by quantitative real-time PCR. Differences between treatments were assessed by Student’s *t* test. * *p* < 0.05 vs. control; ** *p* < 0.01 vs. control; *** *p* < 0.001 vs. control. Sim.: simvastatin.

Thus, MK-7 appeared to act with a similar mechanism of action as statins, such as by inhibiting the cholesterol biosynthetic pathway thereby inducing cholesterol related genes to counteract this action. To further explore this possibility, we determined the mRNA expression levels of LDLR and HMG-CoA reductase by RT-qPCR. As shown in Figure 3, 24 h incubation with MK-7 at 7.5 µM significantly induced both genes to similar extents as the 40 µM simvastatin.

### 3.3. Squalene Counteracts the Effects of MK-7 on LDLR in the Hepatic Cell Line

**Figure 4:**
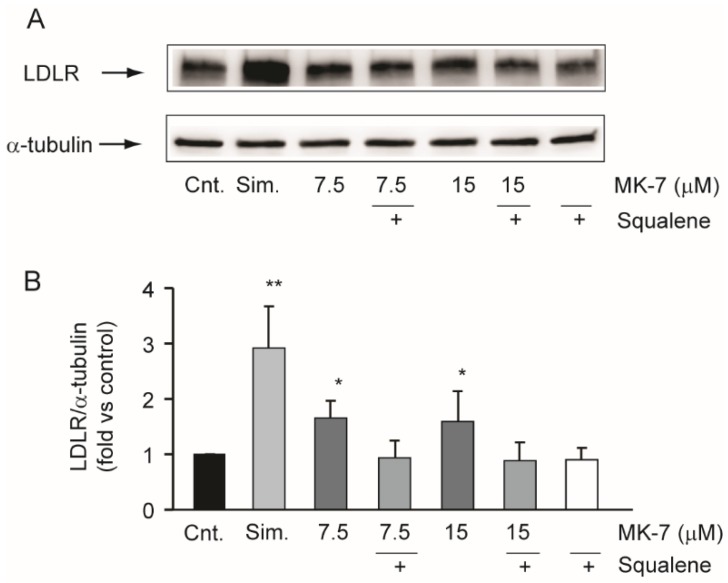
Effect of squalene on MK-7-dependent induction of LDLR. (**A**,**B**) Human hepatoma cells were incubated for 24 h with simvastatin (5 µM) and MK-7 in the presence or absence of squalene (10 µM). LDLR protein expression was evaluated by Western blot analysis. α-tubulin was used as the loading control. (**B**) Densitometric readings were evaluated using ImageLab^TM^ software. Differences between treatments were assessed by Student’s *t* test. * *p* < 0.05 vs. control; ** *p* < 0.01; Sim.: simvastatin.

Starting from the chemical similarity of MK-7 and isoprenoids moieties of the mevalonate pathway (all-*trans* farnesyl-PP and all-*trans* geranylgeranyl-PP) [34], we hypothesized that inhibition of cholesterol biosynthesis by MK-7 was upstream of squalene synthesis. We therefore investigated the effect of squalene on LDLR induction by MK-7. As shown in Figure 4, the effect of MK-7 on LDLR was prevented by the co-incubation with squalene, suggesting that MK-7 was capable of interfering with cholesterol biosynthesis at an enzymatic step upstream of squalene biosynthesis.

### 3.4. MK-7 Reduces the Expression of PCSK9 in the Hepatoma Cell Line

**Figure 5:**
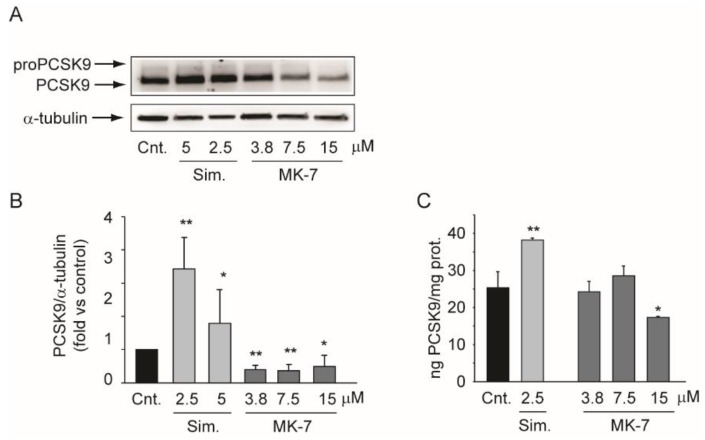
Effect of MK-7 on PCSK9 expression. (**A**) Human hepatoma cells were incubated for 24 h with indicated concentrations of simvastatin and MK-7. PCSK9 protein expression was evaluated by Western blot analysis. α-tubulin was used as the loading control. (**B**) Densitometric readings were evaluated using ImageLab^TM^ software. (**C**) Cells were treated under the same experimental conditions as panel A and the conditioned media were analyzed by ELISA to determine PCSK9 concentrations. The data were normalized for the total cell protein. Differences between treatments were assessed by Student’s *t* test. * *p* < 0.05 vs. control; ** *p* < 0.01 vs. control; Sim.: simvastatin.

Proprotein Convertase Subtilisin/Kexin Type 9 (PCSK9) is a pivotal regulator of cholesterol homeostasis [35]. For this reason, we investigated the effect of MK-7 on its expression in the Huh7 cell line. The intracellular expression of PCSK9 was evaluated by Western blot analysis, while its secretion was evaluated by ELISA after 24 h of incubation with simvastatin or MK-7. By inhibiting cholesterol biosynthesis and activating the sterol responsive element binding protein (SREBP) pathway, simvastatin increased PCSK9 expression at both the intra- and extracellular level (Figure 5) [36,37]. In contrast, under the same experimental conditions, MK-7 significantly suppressed PCSK9 expression (Figure 5A,B) and its secretion (Figure 5C). These results suggested that MK-7 acts according to a different mechanism of action than simvastatin.

## 4. Discussion

Vascular calcification is a common complication in chronic kidney disease (CKD) [38]. However, so far, the complex processes underlying this pathology remain incompletely understood. Consequently, no convincing concepts and treatment options to prevent or reduce the development of vascular calcification are yet available [39]. Although the dysregulation of mineral homeostasis and elevated phosphate levels are considered to be key determinants of vascular calcification in CKD [40], additional key players may strongly contribute, e.g., MGP, which is a powerful inhibitor of tissue calcification. In this work, we tested the hypothesis that diet supplementation with a formulation containing MK-7 (3.5 µg/g of diet), MgCO_3_ (3.7 µg/g of diet), and Sucrosomial^®^ Iron (1mg/g of diet) may have protective role against vascular calcification by turning inactive uncarboxylated MGP to active MGP [15]. In vitro experiments clearly demonstrated effective inhibition of calcium deposition in the extracellular environment. However, we did not observe any variation in vascular calcification in vivo, as determined by histochemical and biochemical analyses. The lack of effect may have been due to the experimental model that was utilized, which induced rapid and massive vascular calcification, or the duration and dose of the treatment. Indeed, we did not observe any significant changes in MGP mRNA levels in the rats fed the supplemented diet. On this regard, the analytical determination of MK-7 from rat plasma revealed that the concentration of MK-7 was equal to 11.9 ± 7.9 ng/mL, which was far below what was utilized in vitro (0.3 mg/mL). This amount of supplementation was decided according to the Italian legislation for nutraceuticals [41].

An interesting observation was that the uremic rats developed a mild, but significant, hypercholesterolemia, and the supplemented diet partially controlled this condition. Starting from this evidence, we set up a series of in vitro experiments aimed at determining the effect of the nutraceutical combination on cholesterol metabolism. We first observed that ethanol extract of the powder present in RenaTris^®^ capsules significantly induced LDLR in the Huh7 cell line, thereby confirming the in vivo evidence relating to cholesterol levels. We then analyzed the effect of MK-7 on cholesterol metabolism in vitro. Interestingly, MK-7 potently inhibited cholesterol biosynthesis in the human hepatocarcinoma cell line. This effect was observed at a concentration above 7.5 µM, which was 50-fold higher than the simvastatin (0.15 µM) [42]. However, unlike simvastatin, MK-7 showed potent and effective inhibitory activity regarding PCSK9 secretion, an effect that may have significantly contributed to the hypocholesterolemic action observed in vivo. A similar effect on cholesterol-related genes was demonstrated with berberine, which prolonged LDLR mRNA stability and therefore its expression while reducing PCSK9 transcription [43,44,45]. However, MK-7 and berberine have completely different chemical structures, therefore it is unlikely that they act via the same intracellular target. In this regard, MK-7 possesses six unsaturated isoprenoid residues linked to a menaquinone moiety. This long carbon tail very closely resembles the chemical structure of squalene, a precursor of cholesterol in the mevalonate pathway [34]. Although HMG-CoA reductase is considered to be the major regulatory enzyme of cholesterol biosynthesis, squalene synthase may play a regulatory function under certain conditions, since it catalyzes the first committed step in cholesterol biosynthesis [34]. Thus, we hypothesized that MK-7 may elicit an inhibitory action on the catalytic activity of squalene synthase in a similar way to lapaquistat [46]. Although a direct inhibitory effect on this enzyme still needs to be determined, we observed complete prevention of the effect of MK-7 on LDLR by co-incubation with squalene. This result suggested that squalene counteracts the effect of MK-7 on cholesterol biosynthesis, therefore, MK-7 inhibits this pathway at an enzymatic step upstream of squalene synthase.

## 5. Conclusions

Taken together, the present work demonstrated the lack of efficacy of a new nutraceutical combination, containing MK-7, magnesium carbonate, and Sucrosomial^®^ Iron, on vascular calcification under uremic conditions. However, we discovered that it possessed hypocholesterolemic action in vivo in uremic rats and identified MK-7 as the main active constituent with cholesterol-inhibition activity. In the future, it will be interesting to study the effect of RenaTris^®^ on cholesterol levels in patients affected by CKD.

## Figures and Tables

**Table 1:** Primer sequence utilized for the qPCR analysis.

Gene	Forward (5′–3′)	Reverse (5′–3′)	Efficiency
*HMGCR*	CTTGTGTGTCCTTGGTATTAGAGCTT	GCTGAGCTGCCAAATTGGA	125%
*LDLR*	TCTATGGAAGAACTGGCGGC	ACCATCTGTCTCGAGGGGTA	93%
*MGP*	GCAGCCCTGTGCTATGAATCT	TTTAGCGTGCCATCTCTGCT	91%
*18S*	CGGCTACCACATCCACGGAA	CCTGAATTGTTATTTTTCGTCACTACC	99%

**Table 2:** Biochemical parameters measured after six weeks of diet treatment. * *p* < 0.05 vs. control diet; ** *p* < 0.01; *** *p* < 0.001 vs. control diet; § *p* < 0.05 vs. uremic diet.

Group	Phosphate (mmol/L)	Ca^2+^ Aorta (mg/g tissue)	Total Cholesterol (mg/dL)	Creatinine (µmol/L)	Iron (µmol/L)
*Control diet*	2.8 ± 0.3	0.28 ± 0.12	63.0 ± 19.0	25.3 ± 2.8	27.5
*Uremic diet*	5.1 ± 1.5 *	2.87 ± 1.76 ***	95.9 ± 8.2 *	206.4 ± 45.0 **	25.8
*Supplemented uremic diet*	4.5 ± 1.4 *	3.28 ± 1.99 ***	77.7 ± 116 *§	241.0 ± 71.7 **	32.0
